# Machine learning-based prediction of difficult laryngoscopy in infants with Pierre Robin sequence using quantitative 3D computed tomography parameters

**DOI:** 10.3389/fneur.2026.1784503

**Published:** 2026-06-24

**Authors:** Danling Hu, Weiwei Cai, Anwen Zheng, ShuaiLi You, Shan Zhong

**Affiliations:** Department of Anesthesiology, Children’s Hospital of Nanjing Medical University, Nanjing, China

**Keywords:** airway assessment, difficult laryngoscopy, machine learning, Pierre Robin sequence, predictive model, three-dimensional computed tomography

## Abstract

**Background:**

Infants with Pierre Robin sequence (PRS) frequently present with difficult laryngoscopic exposure due to mandibular hypoplasia, glossoptosis, and upper airway narrowing. Quantitative three-dimensional computed tomography (3D-CT) enables objective characterization of airway anatomy and may improve preoperative airway assessment. This study aimed to identify key 3D-CT parameters associated with difficult laryngoscopic exposure in infants with PRS and to develop and externally validate machine learning–based predictive models.

**Methods:**

We retrospectively analyzed 214 infants with PRS who underwent mandibular distraction osteogenesis between 2023 and 2024. According to laryngoscopic view, patients were classified into an easy-exposure group (Cormack–Lehane grades I–II) and a difficult-exposure group (grades III–IV). Univariable and multivariable logistic regression analyses were performed to identify independent quantitative 3D-CT predictors. Seven machine learning models were constructed, including logistic regression, support vector machine, random forest, Extra Trees, XGBoost, LightGBM, and AdaBoost. Internal performance was evaluated using five-fold cross-validation, and generalizability was assessed in an independent temporal validation cohort from the same institution (*n* = 80). Model performance was assessed using discrimination, calibration, and decision curve analysis.

**Results:**

Multivariable analysis identified four independent predictors: tongue length (D1) (odds ratio [OR] = 1.058, *p* = 0.005), tongue base–posterior pharyngeal wall distance (D4) (OR = 0.718, *p* < 0.001), sagittal oropharyngeal cross-sectional area (S2) (OR = 0.271, *p* = 0.001), and tongue base–epiglottic angle (A2) (OR = 0.952, *p* = 0.028). Among all models, XGBoost achieved the highest discrimination in the training cohort (AUC = 0.961). However, in the temporal validation cohort from the same institution, the Extra Trees model demonstrated superior generalizability (AUC = 0.876), with an accuracy of 0.812 and an F1-score of 0.805. Calibration analysis indicated excellent agreement between predicted and observed outcomes for the Extra Trees model (Hosmer–Lemeshow test, *p* > 0.999). Decision curve analysis showed a substantial net clinical benefit across threshold probabilities ranging from approximately 10 to 90%.

**Conclusion:**

Quantitative 3D-CT parameters reflecting tongue morphology and oropharyngeal airway dimensions are clinically relevant predictors of difficult laryngoscopic exposure in infants with PRS. The Extra Trees model showed promising performance in temporal validation within a single-center cohort.

## 1 Background

Pierre Robin sequence (PRS) is a congenital craniofacial anomaly characterized by micrognathia, glossoptosis, and varying degrees of upper airway obstruction (1). Approximately 85% of affected infants have an associated cleft palate, and the estimated incidence ranges from 1 per 8,000 to 1 per 14,000 births, with no significant sex predominance (2). Mandibular hypoplasia and posterior displacement of the tongue markedly reduce the oropharyngeal airway area (3, 4), predisposing the upper airway to inspiratory collapse and leading to varying degrees of respiratory and feeding difficulties. When conservative measures such as positional therapy, nasopharyngeal airway placement, or continuous positive airway pressure (CPAP) fail, surgical intervention becomes the primary approach to relieving upper airway obstruction. Tongue–lip adhesion (TLA) was historically employed to anteriorly reposition the tongue and enlarge the airway. However, its effectiveness is often limited by recurrent tongue posteriorization and scar contracture. Consequently, a proportion of infants ultimately still require tracheostomy or long-term gastrostomy dependence (5). Over the past two decades, mandibular distraction osteogenesis (MDO) has increasingly supplanted tongue–lip adhesion as the preferred surgical treatment for severe airway obstruction in infants with PRS. By gradually advancing the mandible, MDO effectively relieves glossoptosis, enlarges the upper airway, and has been shown to significantly improve respiratory function and feeding outcomes (6–9). Nevertheless, MDO is performed under general anesthesia, and securing the airway during induction remains a major anesthetic challenge.

Infants with PRS frequently present with difficult laryngoscopic exposure owing to micrognathia, restricted oral aperture, and posterior tongue positioning. Failed or prolonged intubation in this vulnerable population may lead to hypoxemia, airway trauma, or emergent surgical airway intervention. Therefore, accurate preoperative identification of a potentially difficult airway is of critical importance for anesthetic planning and patient safety (10). Traditional methods for predicting difficult laryngoscopic exposure rely largely on external anatomical or clinical bedside assessments, such as the Mallampati grading (MPG), interincisor gap (IIG), thyromental distance (TMD), and mandibular angle (11). However, in neonates and infants, these indicators often fail to accurately characterize true intubation difficulty due to the unique anatomical proportions of the upper airway, limited cooperation, and the inherently low objectivity of available assessment tools (12, 13).

Advances in three-dimensional computed tomography (3D-CT) have enabled precise quantitative assessment of upper airway anatomy, including volumetric measurements, cross-sectional areas, and spatial relationships of key airway structures (14, 15). The clinical application of CT-based assessment should be interpreted with caution, as its routine use may be limited by radiation exposure, cost, and accessibility. Therefore, such approaches are most applicable in patients who have already undergone CT imaging as part of standard preoperative evaluation. Although several studies have indicated that certain CT-derived parameters are associated with difficult laryngoscopic exposure in infants with PRS, predictive models specifically tailored for preoperative assessment in this population remain limited. Most existing research has focused on single parameters or isolated anatomical features, lacking integrated quantitative modeling and external validation (10, 16, 17). In addition, difficult laryngoscopic exposure in infants with PRS is likely influenced by complex interactions among multiple anatomical parameters rather than a single isolated feature. Traditional statistical models, such as logistic regression, may be limited in capturing nonlinear relationships and high-order interactions among variables. In contrast, machine learning approaches provide flexible frameworks capable of handling high-dimensional data and modeling complex, nonlinear patterns, which may improve predictive accuracy in this context (18, 19).

Therefore, this study aimed to (1) identify key preoperative 3D-CT parameters associated with difficult laryngoscopic exposure in infants with PRS, and (2) develop and perform temporal validation using an independent cohort from the same institution to validate a series of machine learning–based predictive models using these quantitative imaging features. By establishing an objective and quantitative predictive framework, this study aims to support improved preoperative risk assessment and perioperative airway management in infants undergoing MDO.

## 2 Materials and methods

### 2.1 Study design and population

This single-center retrospective cohort study was conducted at Nanjing Children’s Hospital affiliated with Nanjing Medical University and was approved by the institutional Ethics Committee (Approval No. 202503051-1). Given the retrospective design, the requirement for written informed consent from the guardians was waived.

A total of 214 infants diagnosed with Pierre Robin sequence (PRS) who underwent mandibular distraction osteogenesis (MDO) between January 2024 and December 2024 were screened for eligibility. The inclusion criteria were: (1) a clinical diagnosis consistent with PRS (micrognathia and glossoptosis, with or without cleft palate); (2) radiological evidence of upper airway narrowing on preoperative three-dimensional computed tomography (3D-CT); and (3) availability of complete laryngoscopic grading data. Exclusion criteria included: (1) the presence of additional craniofacial anomalies or severe cardiopulmonary disease; and (2) incomplete clinical or imaging records. All 3D-CT images were obtained for routine clinical preoperative assessment to guide surgical planning for mandibular distraction osteogenesis. No additional imaging was performed for research purposes.

### 2.2 Clinical and imaging data

All infants underwent craniofacial CT scanning under a standardized sedation protocol (oral chloral hydrate, 50 mg/kg). To minimize motion artifacts, all infants were scanned under standardized sedation conditions. Scans were performed in the supine position with the head maintained in a neutral midline orientation. Imaging was acquired using a Philips Brilliance iCT scanner, with the scan range extending from the nasopharyngeal roof to the subglottic region. The acquisition parameters were as follows: tube voltage 100 kV; automatic tube current modulation (DRI = 20); pitch 0.4; rotation time 0.5 s; and a 512 × 512 matrix. Images were reconstructed using both bone and standard kernels with a slice thickness of 1 mm and an interslice spacing of 1 mm, ensuring high-resolution datasets suitable for three-dimensional airway analysis.

The CT parameters evaluated in this study were selected based on established anatomical mechanisms contributing to difficult laryngoscopic exposure in infants with PRS, including indicators of glossoptosis (e.g., D4), mandibular development (e.g., D5–D8), oropharyngeal patency (e.g., S2, S3, V1), and angular measurements influencing laryngoscope blade placement and glottic visualization (e.g., A1, A2) (16, 20–22). DICOM-format craniofacial CT scans were imported into 3D Slicer (version 5.8.1) for three-dimensional reconstruction of the maxillofacial skeleton and upper airway. The midsagittal plane was determined by adjusting orthogonal slices to simultaneously pass through the sella midpoint, anterior nasal spine, and posterior midpoint of the foramen magnum, and was locked after confirming bilateral symmetry (23).

**Figure 1:**
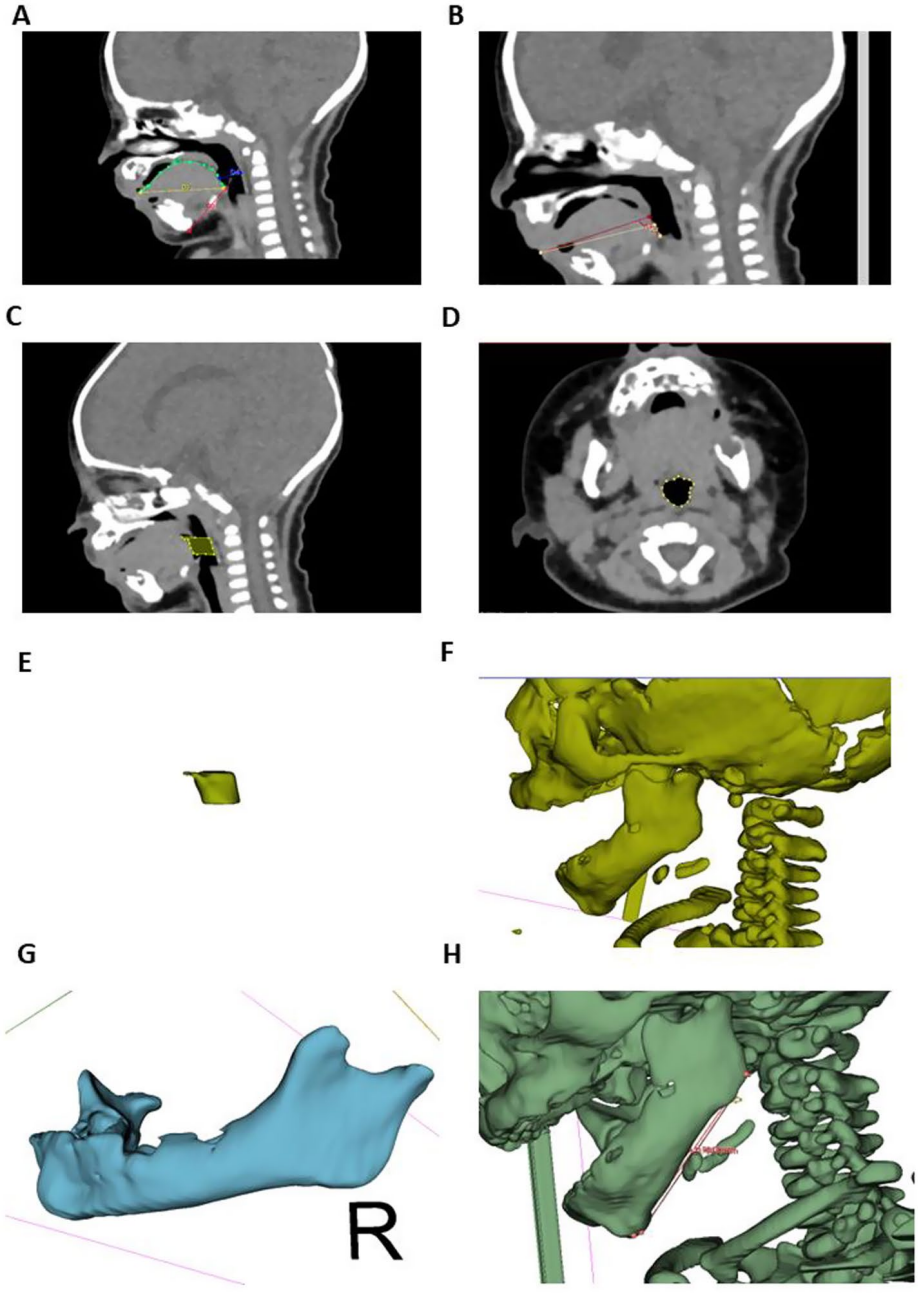
Schematic illustration of quantitative 3D-CT parameters. **(A)** Midsagittal view illustrating tongue-related measurements (D1–D4). **(B)** Sagittal view of the pharyngeal airway space and angular parameters (A1, A2). **(C)** Measurement of sagittal oropharyngeal cross-sectional area (S2). **(D)** Identification of minimal oropharyngeal cross-sectional area (S3) on axial images. **(E)** Three-dimensional reconstruction for measurement of oropharyngeal volume (V1). **(F–H)** Three-dimensional reconstruction of craniofacial structures, including airway, mandible, and linear mandibular dimensions (D5–D8). D, distance; S, cross-sectional area; V, volume; A, angle.

On this standardized midsagittal plane, the following quantitative measurements were obtained: D1, curved surface length of the tongue body (mm); D2, linear distance from pogonion to the base of the epiglottis (mm); D3, linear distance from the tongue tip to the epiglottic base (mm); D4, shortest distance from the tongue base to the posterior pharyngeal wall (mm); D5–D8, mandibular body and ramus lengths on the right and left sides (mm); S1–S3,sagittal cross-sectional areas of the tongue and oropharyngeal airway and the minimal oropharyngeal cross-sectional area (cm^2^); V1, oropharyngeal cavity volume (mm^3^). Angular parameters were defined as follows: A1, the angle formed by the geometric midpoint of the stomion line, the free margin of the soft palate, and the epiglottic base; and A2, the angle formed by the geometric midpoint of the stomion line, the tongue base, and the epiglottic base. All parameters are referred to as D1–D8, S1–S3, V1, A1, and A2 in subsequent analyses, with illustrative diagrams provided in Figure 1.

All imaging measurements were performed with the assessor blinded to the laryngoscopic outcomes. Each parameter was independently measured three times by the same investigator, and the average of the three measurements was used for statistical analysis. The repeatability of the measurements was evaluated using the intraclass correlation coefficient (ICC). ICC (3,1) was computed to assess the reliability of single measurements, while ICC (3,3) was used to assess the reliability of the average of three measurements. Both indices were derived from a two-way mixed-effects model with absolute-agreement definition. ICC values <0.50 were interpreted as poor reliability, 0.50–0.75 as moderate, 0.75–0.90 as good, and >0.90 as excellent.

### 2.3 Anesthesia protocol

**Figure 2:**
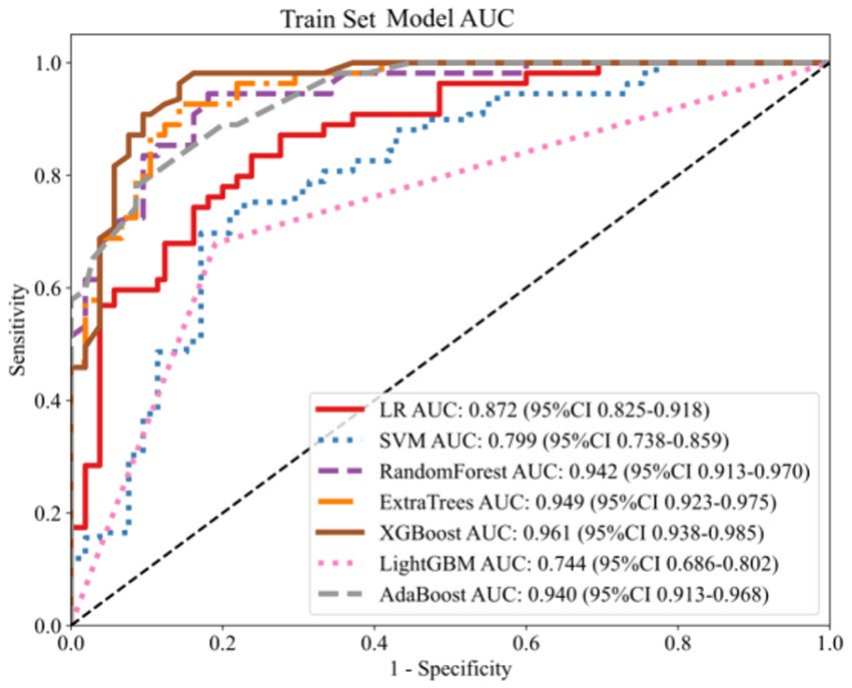
Receiver operating characteristic (ROC) curves of seven machine learning models in the training cohort.

Following completion of the CT scan, each infant was positioned supine. After adequate preoxygenation, initial anesthesia induction was performed intravenously using propofol and sufentanil. Manual mask ventilation was then immediately attempted to assess the ease of ventilation. If ventilation was satisfactory, direct laryngoscopy was performed in the standard sniffing position to evaluate glottic visualization, and the Cormack–Lehane (CL) grade was recorded (Figure 2).

Once it was confirmed that the infant had no difficulty with mask ventilation and the airway could be safely maintained, cisatracurium was administered to achieve adequate neuromuscular relaxation, followed by endotracheal intubation. In cases of suboptimal glottic exposure under direct laryngoscopy, BURP maneuver or fiberoptic bronchoscope–assisted intubation was employed as appropriate.

After successful intubation, additional propofol, opioids, and neuromuscular blockers were administered according to institutional practice to complete anesthesia induction. Throughout the procedure, continuous monitoring of blood pressure, heart rate, and SpO₂ was performed, and the rate of drug administration was adjusted based on hemodynamic and respiratory status to avoid hypotension or respiratory depression.

The infant was then transferred to the operating room, where anesthesia was maintained with sevoflurane at 1.5–2.0 MAC, supplemented with low-dose opioids as needed for analgesia. Standard intraoperative monitoring included electrocardiography, noninvasive blood pressure, pulse oximetry, and end-tidal carbon dioxide (PetCO₂), with PetCO₂ maintained at 35–45 mmHg to ensure respiratory and hemodynamic stability.

### 2.4 Outcomes and definitions

Airway exposure difficulty was assessed using the Cormack–Lehane (CL) grading obtained during the first laryngoscopic evaluation in surgery. CL grade I indicated full visualization of the glottis; grade II, partial visualization of the glottis; grade III, visualization of the epiglottis only; and grade IV, no visualization of either the glottis or the epiglottis. The primary outcome was defined as the initial CL grade recorded before the use of any adjunctive maneuvers. Although the BURP maneuver could be applied when clinically necessary for airway safety, its use did not alter the recorded outcome.

In accordance with previous literature and clinical convention, CL grades I–II were classified as the “easy exposure group” (Group A), and CL grades III–IV were classified as the “difficult exposure group” (Group B). This dichotomized classification served as the primary endpoint of the study. This dichotomized classification was chosen as the primary endpoint because CL grade is a well-established surrogate for difficult intubation and is strongly associated with an increased risk of adverse perioperative events, such as hypoxemia and airway trauma, in the PRS population. Furthermore, it allows for direct comparability with existing literature on preoperative airway assessment in infants.

### 2.5 Statistical analysis

Continuous variables are presented as mean ± standard deviation or median (interquartile range), while categorical variables are described as counts and percentages. Differences between groups were assessed using independent samples t-tests or the chi-squared test. Independent predictors used for machine learning model training were identified via univariate and multivariate logistic regression analyses. Multicollinearity among variables was assessed using variance inflation factors (VIF), and all variables showed VIF values < 5, indicating no significant multicollinearity. All statistical analyses were conducted using Python (version 3.9.0) and SPSS (version 26.0). A two-tailed *p*-value < 0.05 was considered statistically significant.

### 2.6 Machine learning model development and evaluation

The dataset consisted of 214 infants with PRS who underwent mandibular distraction osteogenesis at our institution between 2023 and 2024. No explicit feature scaling or normalization was applied. Tree-based models are generally insensitive to feature scaling; however, we acknowledge that scaling may improve the performance of certain algorithms such as support vector machines. To ensure robust model development and mitigate data leakage, we implemented a nested cross-validation (NCV) framework. Specifically, a 5-fold outer loop was used for model evaluation, while an inner loop was used for feature selection and hyperparameter optimization.

In addition, we collected data from 80 infants who underwent mandibular distraction osteogenesis at our institution in 2025 to form an independent temporal validation set for evaluating model performance over time within the same institution. Within each training fold of the outer loop, feature selection was performed using Least Absolute Shrinkage and Selection Operator (LASSO) regression. Both the regularization parameter (*λ*) of LASSO and the hyperparameters of each machine learning model were optimized exclusively within the inner cross-validation loop using grid search. This design ensured that feature selection and model tuning were conducted using only training data, thereby avoiding information leakage. We constructed and compared seven common machine learning models: Logistic Regression (LR), Support Vector Machine (SVM), Random Forest (RF), Extremely Randomized Trees (ExtraTrees), eXtreme Gradient Boosting (XGBoost), Light Gradient Boosting Machine (LightGBM), and Adaptive Boosting (AdaBoost). All models were implemented using the Python Scikit-learn library (version 1.0.1). Hyperparameter tuning was performed using grid search within the inner loop of the nested cross-validation framework. The search space for each model was predefined and included key parameters such as regularization strength (for logistic regression and SVM), number of estimators and maximum tree depth (for tree-based models), and learning rate (for boosting algorithms). All hyperparameters were optimized exclusively on the training data within the inner loop to avoid information leakage.

Model performance was assessed using the area under the receiver operating characteristic curve (AUC), sensitivity, specificity, precision, and F1-score. To further evaluate the accuracy of the optimal model’s predicted probabilities, we plotted calibration curves and employed the Hosmer–Lemeshow (H–L) test to quantify the goodness-of-fit; a non-significant *p*-value (*p* > 0.05) indicated good agreement between the predicted and observed values. Furthermore, Decision Curve Analysis (DCA) was performed to evaluate the net benefit of the model in clinical decision-making, thereby demonstrating its potential clinical utility. To improve the interpretability of the final machine learning model, SHAP (Shapley Additive Explanations) analysis was performed to quantify the contribution of each feature to model predictions (24). SHAP values were calculated based on the trained ExtraTrees model using the TreeExplainer algorithm, which is specifically optimized for tree-based models. All SHAP analyses were conducted using the SHAP Python package.

## 3 Results

### 3.1 Univariate and multivariate logistic regression analysis of 3D CT metrics

To identify independent imaging predictors of difficult laryngoscopic exposure, univariate and multivariate logistic regression analyses were conducted in the training cohort. As shown in Table 1, univariate analysis demonstrated significant associations between difficult laryngoscopy and multiple quantitative 3D-CT–derived parameters, including tongue curved length (D1), mandible–epiglottis distance (D2), retrolingual space (D4), mandibular body lengths (D5 and D7), mandibular ramus height (D6), sagittal oropharyngeal cross-sectional area (S2), minimal oropharyngeal area (S3), oropharyngeal volume (V1), as well as the oropharyngeal angle (A1) and glosso-epiglottic angle (A2).

**Table 1:** Univariate and multivariate logistic regression analyses of quantitative 3D-CT parameters in the training cohort.

Parameter	Univariate logistic regression		*p*-value	Multivariate logistic regression		*p*-value
	OR	95%CI		OR	95%CI	
D1 (mm)	1.029	1.004–1.055	0.021*	1.058	1.017–1.100	0.005*
D2 (mm)	0.952	0.909–0.998	0.042*	0.983	0.909–1.062	0.662
D3 (mm)	0.998	0.962–1.035	0.911			
D4 (mm)	0.604	0.586–0.753	<0.001*	0.718	0.603–0.854	<0.001*
D5 (mm) (right)	0.939	0.907–0.973	<0.001*	0.980	0.852–1.128	0.782
D6 (mm) (right)	0.914	0.864–0.967	0.002*	0.916	0.831–1.008	0.073
D7 (mm) (left)	0.940	0.907–0.973	<0.001*	0.996	0.865–1.147	0.956
D8 (mm) (left)	0.959	0.906–1.015	0.147			
S1 (cm^2^)	0.989	0.877–1.115	0.857			
S2 (cm^2^)	0.234	0.122–0.449	<0.001*	0.271	0.125–0.587	0.001*
S3 (cm^2^)	0.171	0.084–0.348	<0.001*	0.416	0.161–1.074	0.070
V1 (mm^3^)	1.000	0.999–1.000	<0.001*	1.000	1.000–1.000	0.947
A1 (°)	0.956	0.936–0.976	<0.001*	1.020	0.971–1.072	0.422
A2 (°)	0.928	0.907–0.950	<0.001*	0.952	0.911–0.995	0.028*

OR, Odds Ratio; CI, Confidence Interval; *p < 0.05. No significant multicollinearity was observed among variables (all VIF < 5).

Variables showing significance in the univariate analysis were entered into the multivariate logistic regression model. Four parameters emerged as independent predictors: greater tongue length (D1) (OR = 1.058, 95% CI: 1.017–1.100, *p* = 0.005), a narrower retrolingual space (D4) (OR = 0.718, 95% CI: 0.603–0.854, *p* < 0.001), a smaller sagittal oropharyngeal area (S2) (OR = 0.271, 95% CI: 0.125–0.587, *p* = 0.001), and a smaller glosso-epiglottic angle (A2) (OR = 0.952, 95% CI: 0.911–0.995, *p* = 0.028) (Table 1). These four independent predictors were subsequently used as input features for machine learning model development.

### 3.2 Machine learning model performance

The outcome distribution in the cohorts was as follows: In the training cohort (*n* = 214), there were 105 (449%) patients in the easy exposure group and 109 (51%) in the difficult exposure group. In the independent temporal validation cohort (*n* = 80), there were 41 (51%) patients in the easy exposure group and 39 (49%) in the difficult exposure group (Table 2).

**Table 2:** Performance metrics of the seven machine learning models in the training and independent test sets.

Set	Model_name	Accuracy	AUC	95% CI	Sensitivity	Specificity	PPV	NPV	Precision	Recall	F1	Threshold
Train	LR	0.799	0.872	0.8254–0.9181	0.835	0.762	0.784	0.816	0.784	0.835	0.809	0.467
	SVM	0.762	0.799	0.7384–0.8588	0.697	0.829	0.809	0.725	0.809	0.697	0.749	0.649
	Random Forest	0.883	0.942	0.9130–0.9701	0.945	0.819	0.844	0.935	0.844	0.945	0.892	0.469
	Extra Trees	0.893 (0.8520–0.9341)	0.949	0.9226–0.9750	0.927 (0.8935–0.9616)	0.857	0.871	0.918	0.871	0.927	0.898	0.501
	XGBoost	0.911	0.961	0.9376–0.9846	0.963	0.857	0.875	0.957	0.875	0.963	0.917	0.45
	Light GBM	0.743	0.744	0.6862–0.8022	0.679	0.81	0.787	0.708	0.787	0.679	0.729	0.562
	AdaBoost	0.846	0.94	0.9133–0.9677	0.78	0.914	0.904	0.8	0.904	0.78	0.837	0.513
Test	LR	0.787	0.862	0.7853–0.9395	0.718	0.854	0.824	0.761	0.824	0.718	0.767	0.618
	SVM	0.787	0.849	0.7656–0.9329	0.821	0.756	0.762	0.816	0.762	0.821	0.79	0.511
	Random Forest	0.738	0.77	0.6651–0.8746	0.795	0.683	0.705	0.778	0.705	0.795	0.747	0.447
	Extra Trees	0.812 (0.724–0.8952)	0.876	0.8014–0.9497	0.795 (0.6874–0.8907)	0.829	0.816	0.81	0.816	0.795	0.805	0.525
	XGBoost	0.8	0.823	0.7271–0.9189	0.769	0.829	0.811	0.791	0.811	0.769	0.789	0.448
	Light GBM	0.688	0.684	0.5846–0.7844	0.564	0.805	0.733	0.66	0.733	0.564	0.638	0.562
	AdaBoost	0.762	0.801	0.7027–0.9002	0.692	0.829	0.794	0.739	0.794	0.692	0.74	0.504

The distribution of outcome classes is reported in the Results section.

**Figure 3:**
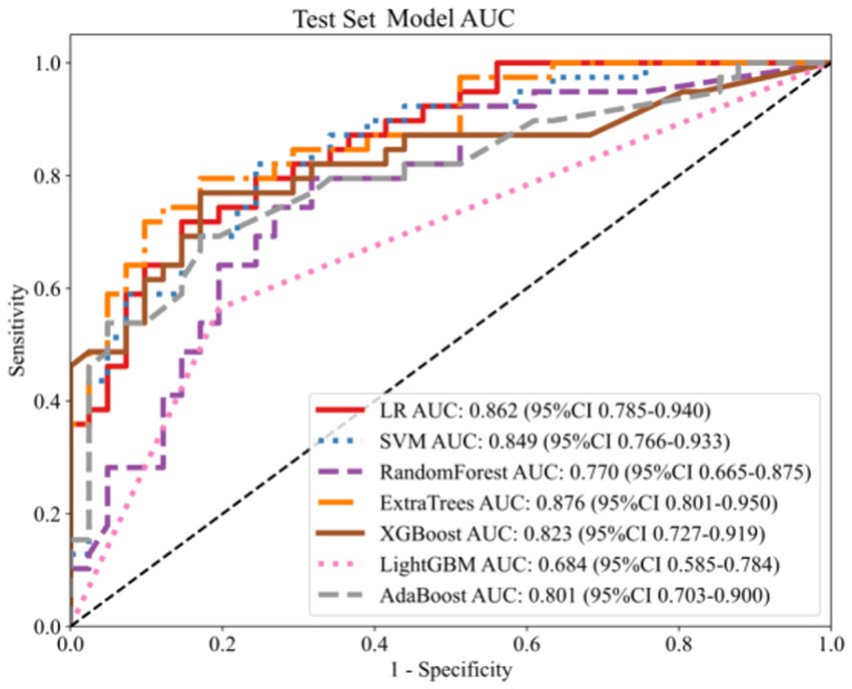
Receiver operating characteristic (ROC) curves of seven machine learning models in the independent temporal validation cohort.

When evaluated in the independent temporal validation cohort collected in 2025, all models showed a modest decline in performance, reflecting the challenge of maintaining model performance over temporally distinct data. Among them, the ExtraTrees model exhibited the most favorable performance, achieving the highest Accuracy (0.812) and AUC (0.876, 95% CI: 0.8014–0.9497), together with balanced Sensitivity (0.795), Specificity (0.829), and an F1-score of 0.805. Logistic Regression and XGBoost also maintained robust performance, with AUCs of 0.862 and 0.823, and Accuracies of 0.787 and 0.800, respectively. In contrast, LightGBM consistently yielded the lowest metrics across both cohorts (Figure 3). Given its superior balance between discrimination and temporal robustness in the validation set, the ExtraTrees algorithm was selected as the final prediction model for further evaluation and potential clinical application.

**Figure 4:**
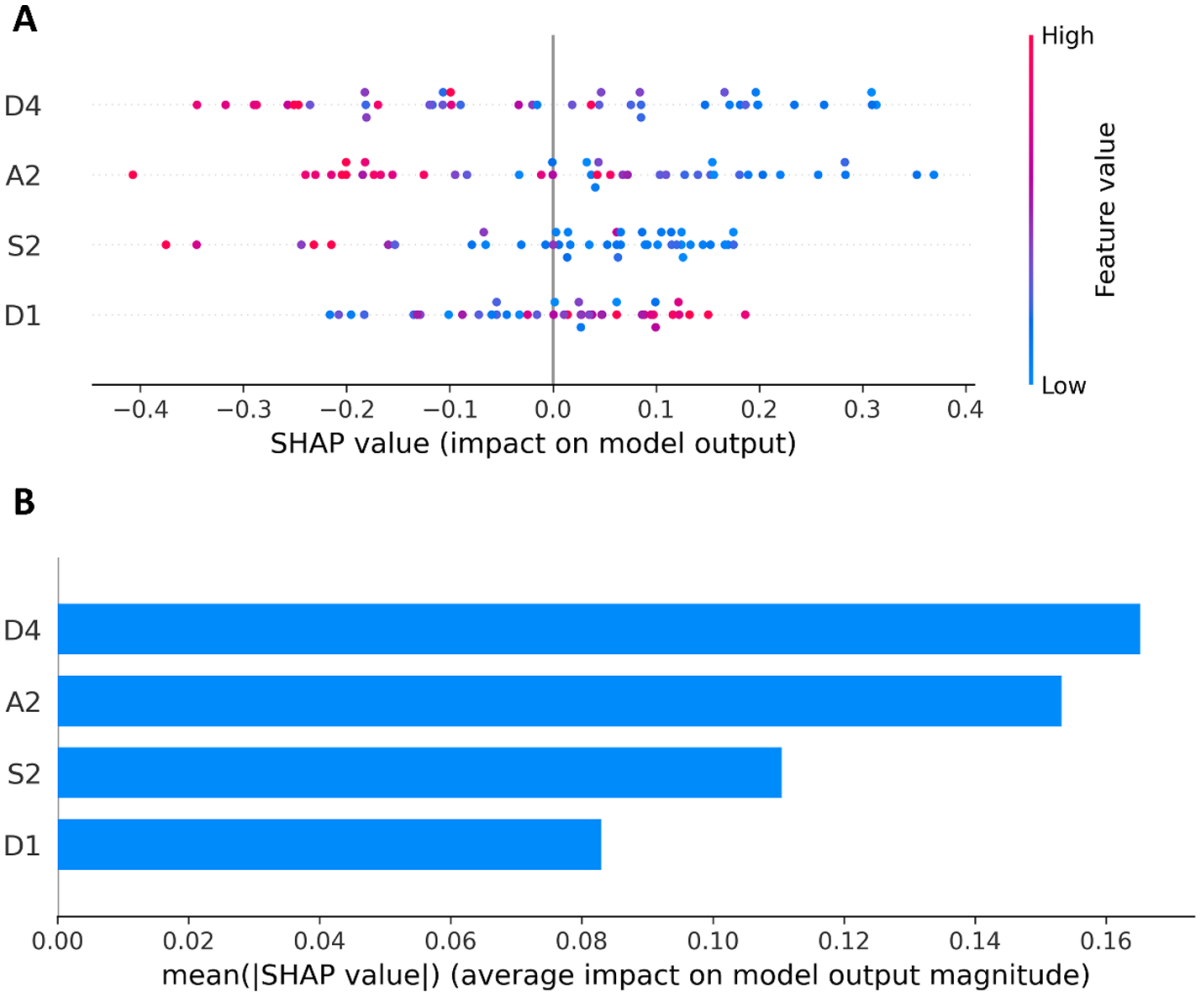
SHAP interpretation of the ExtraTrees model. **(A)** The summary plot (top) illustrates the distribution of SHAP values for each feature, with color indicating feature magnitude. Lower D4, S2, and A2 values were associated with increased risk, whereas higher D1 values contributed to higher predicted risk. **(B)** The bar plot (bottom) shows the mean absolute SHAP values, indicating that D4 had the greatest contribution to model predictions, followed by A2, S2, and D1.

To enhance the interpretability of the final ExtraTrees model, SHAP (Shapley Additive Explanations) analysis was performed to quantify the contribution of each feature to model predictions. The SHAP summary plot (Figure 4) demonstrated that all four selected features contributed to the prediction of difficult laryngoscopic exposure, with varying magnitudes. Among them, retrolingual space (D4) exhibited the greatest impact, followed by the glosso-epiglottic angle (A2), sagittal oropharyngeal area (S2), and tongue length (D1). In terms of directionality, lower values of D4, S2, and A2 were associated with increased risk, whereas higher D1 values contributed positively to the prediction of difficult laryngoscopy. Importantly, the SHAP-derived feature importance ranking and effect directions were consistent with the multivariate logistic regression results, supporting the robustness and biological plausibility of these predictors.

### 3.3 Calibration assessment

**Figure 5:**
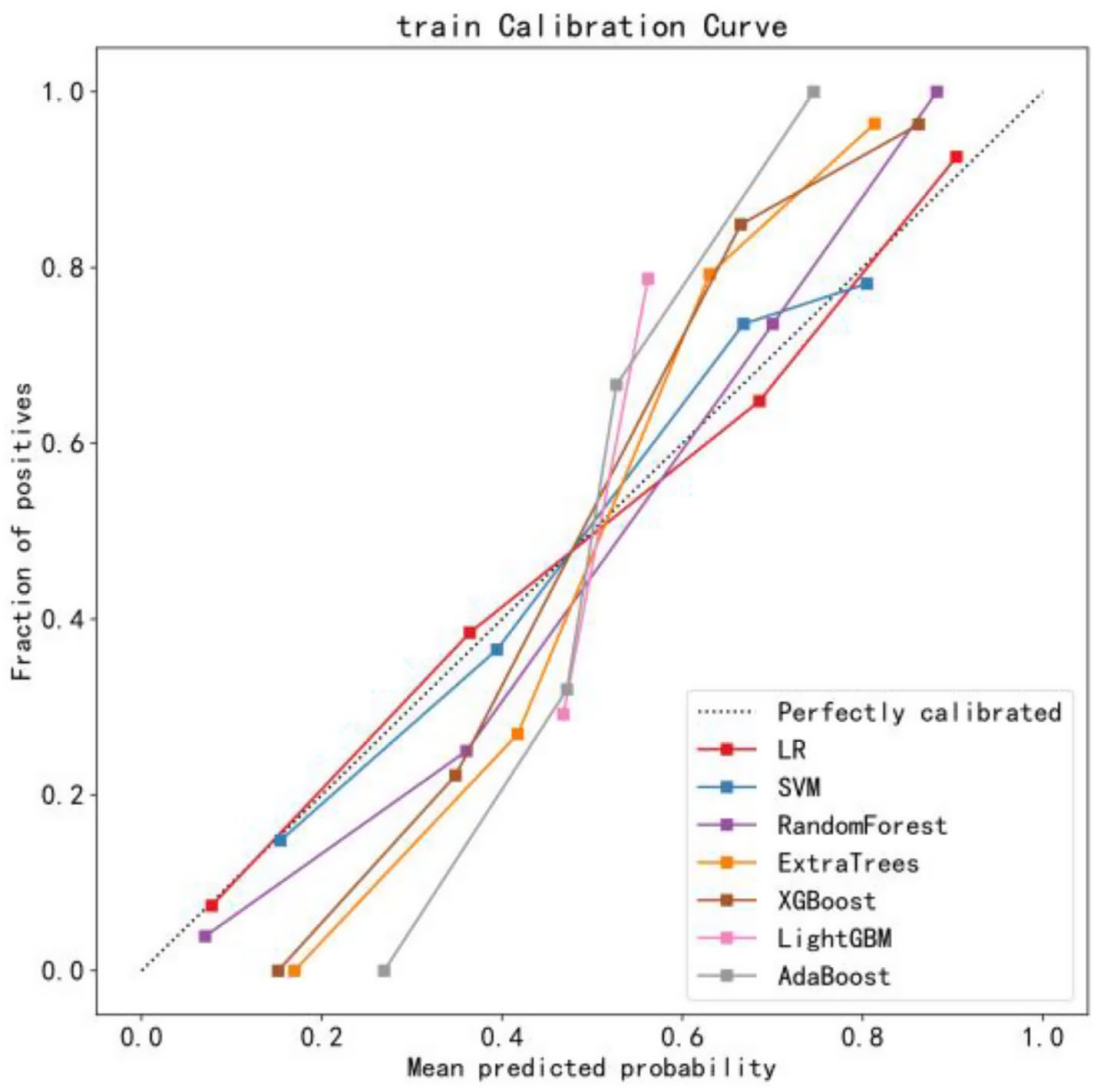
Calibration curves of seven machine learning models in the training cohort.

**Figure 6:**
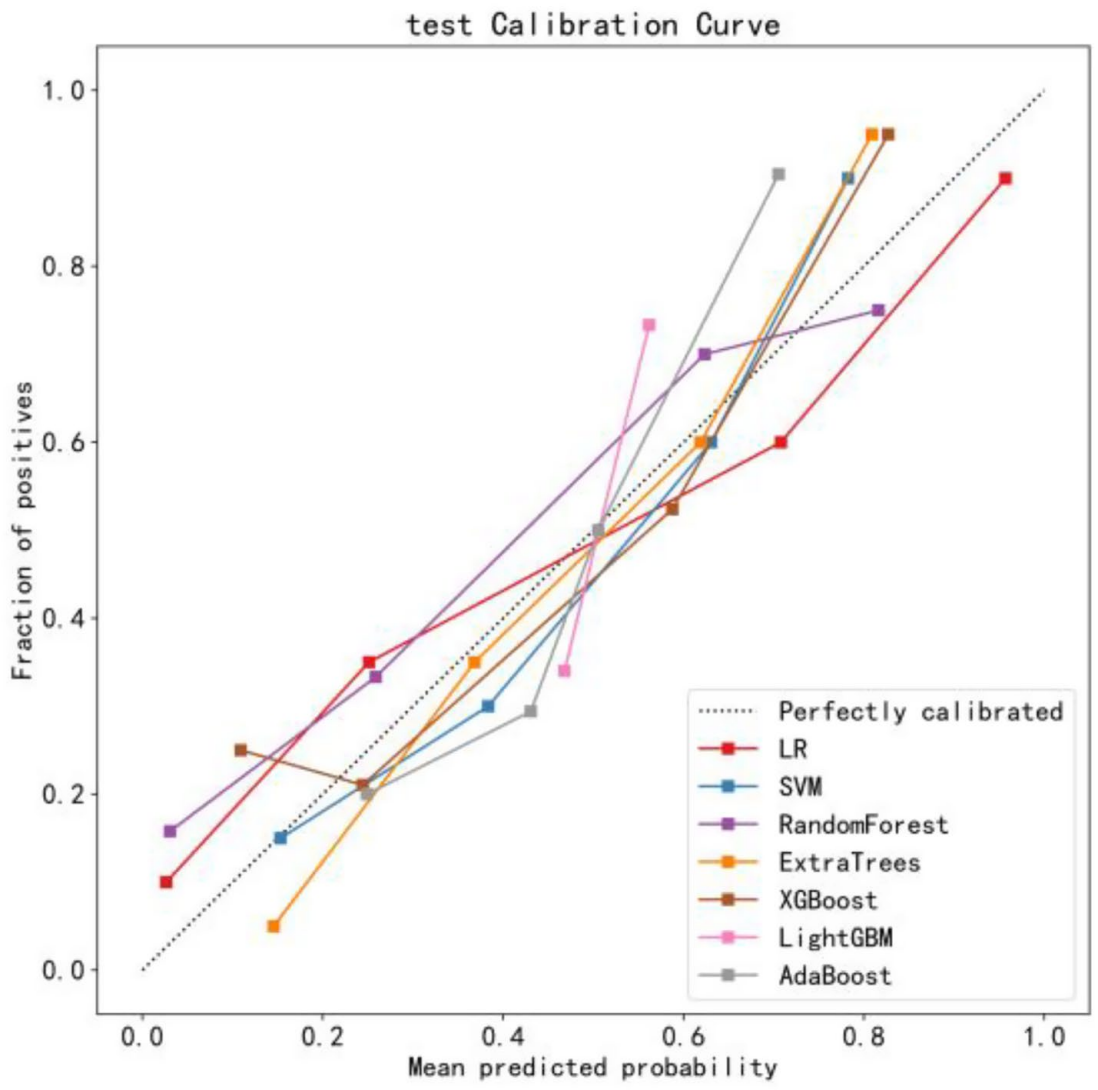
Calibration curves of seven machine learning models in the independent temporal validation cohort.

The calibration of all machine learning models, assessed using the Hosmer--Lemeshow (H-L) goodness-of-fit test, demonstrated good agreement between predicted and observed probabilities in both the training and temporal validation cohorts. Across all models, the H–L test yielded non-significant *p*-values (all *p* > 0.05), indicating no statistically significant deviation from observed outcomes. The final selected ExtraTrees model showed good calibration in the temporal validation cohort (H–L statistic = 0.138, *p* > 0.999). However, this result should be interpreted with caution given the relatively small sample size, and calibration performance was therefore also assessed using calibration curves (Figures 5, 6).

### 3.4 Decision curve analysis

**Figure 7:**
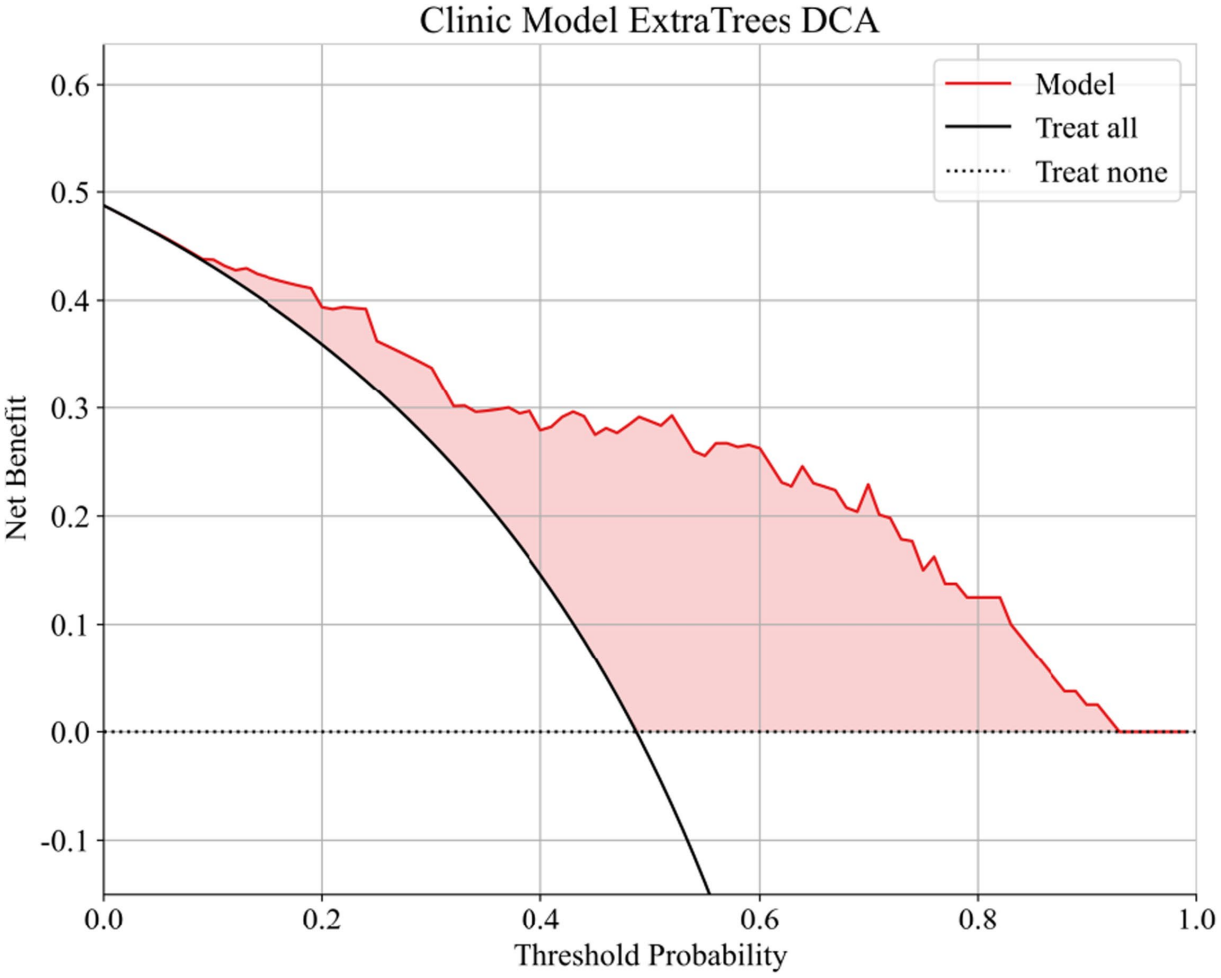
Decision curve analysis (DCA) of the final ExtraTrees prediction model.

Decision curve analysis showed that the ExtraTrees model provided a consistently higher net clinical benefit compared with both the “treat-all” and “treat-none” strategies across a wide range of threshold probabilities, approximately from 10 to 90%. These findings support the potential clinical utility of the model for preoperative prediction of difficult laryngoscopic exposure in infants with Pierre Robin sequence (Figure 7).

## 4 Discussion

Infants with Pierre Robin sequence (PRS) commonly present with varying degrees of upper airway obstruction due to micrognathia, glossoptosis, and oropharyngeal narrowing, making them a prototypical difficult airway population in pediatric anesthesia (25, 26). Compared with older children and adults, PRS infants exhibit pronounced anatomical variability, particularly in mandibular hypoplasia severity, tongue morphology, and patterns of oropharyngeal collapse, all of which markedly influence laryngoscopic visualization (27). Given the inherently small airway and limited procedural space during anesthesia, failure to accurately identify high-risk individuals preoperatively may result in severe peri-induction complications, including hypoxemia and failed intubation (28). Thus, objective and precise preoperative airway assessment is essential for optimizing anesthetic management in this population.

Traditional bedside airway assessments, such as Mallampati classification, thyromental distance, and mandibular angle measurement, rely largely on external anatomical features or two-dimensional imaging, which inadequately capture the complex three-dimensional airway anatomy in PRS infants (29). With recent advances in imaging technology, three-dimensional reconstruction of the upper airway has become feasible. Three-dimensional computed tomography (3D-CT) enables precise quantification of the spatial relationships among the tongue, oropharynx, and hypopharynx in a noninvasive manner, providing an objective foundation for morphological studies of difficult airways. Compared with two-dimensional imaging, 3D-CT can more intuitively demonstrate the extent and direction of airway collapse and allows comprehensive assessment of airway patency through multiple quantitative parameters, including cross-sectional areas, angles, distances, and volumes (14, 30). In this study, quantitative 3D-CT analysis was employed to comprehensively characterize upper airway anatomy in infants with PRS and to elucidate its association with difficult laryngoscopic exposure. Among the numerous structural parameters evaluated, tongue curved length (D1), retrolingual space (D4), sagittal oropharyngeal cross-sectional area (S2), and the glosso-epiglottic angle (A2) were identified as independent predictors, highlighting the multifactorial nature of airway obstruction in this population. Leveraging these key features, seven machine learning models were developed, with the ExtraTrees algorithm demonstrating the most favorable performance—including superior discrimination, excellent calibration, and strong temporal generalizability in an independent 2025 validation cohort. Decision curve analysis further confirmed the model’s substantial clinical net benefit across a broad range of threshold probabilities. Collectively, these findings support the ExtraTrees model as an objective, reproducible, and may serve as a promising and objective tool to assist preoperative risk assessment.

Abnormalities in tongue morphology and upper airway structure constitute the fundamental anatomical basis for difficult laryngoscopic exposure in infants with PRS (22). An increased tongue curved length (D1) indicates tongue elongation, which may predispose to posterior tongue displacement and accentuated posterior pharyngeal wall collapse, thereby limiting glottic visualization. A reduced retrolingual space (D4) and a smaller glosso-epiglottic angle (A2) reflect restricted space between the tongue base and epiglottis as well as posterior displacement of the upper airway. Meanwhile, a decreased sagittal oropharyngeal area (S2) directly represents the degree of airway narrowing and correlates closely with epiglottic exposure. Collectively, these findings suggest that difficult laryngoscopic exposure in PRS results from a synergistic imbalance of multiple structural abnormalities—including tongue elongation, posterior tongue collapse, posterior pharyngeal wall approximation, and reduced sagittal airway area. This study provides quantitative imaging evidence elucidating the pathophysiological mechanisms underlying difficult airway formation in PRS infants.

Machine learning models are inherently capable of managing high-dimensional, nonlinear feature spaces and identifying complex interactions among multiple variables, making them particularly suitable for medical imaging prediction tasks characterized by intricate anatomical structures and strongly correlated parameters (31, 32). In this study, all seven machine learning algorithms achieved solid predictive performance. Notably, the ExtraTrees model demonstrated the highest level of stability, a finding that may be attributed to its “extremely randomized” decision-tree architecture, which naturally mitigates overfitting—especially in datasets with relatively small sample sizes and substantial feature interdependence. In the external temporal validation cohort, the ExtraTrees model maintained a high AUC along with robust sensitivity and specificity, and demonstrated good calibration performance (H–L test *p* > 0.999), indicating that its estimated probabilities for difficult airway risk are highly reliable. It should be noted that the H–L test yielded a high *p*-value in the validation cohort. While this suggests good agreement, it should be interpreted with caution, as the H–L test may be overly conservative in relatively small samples. Therefore, further validation in larger, multi-center cohorts is warranted to confirm model calibration and exclude potential overfitting. Furthermore, decision curve analysis showed that ExtraTrees consistently provided greater net clinical benefit than either the “treat-all” or “treat-none” strategies across a wide threshold probability range (10–90%), underscoring its potential utility in real-world anesthetic decision-making. To improve interpretability, SHAP analysis was applied to the final ExtraTrees model. The results showed that D4 contributed the most to model predictions, followed by A2, S2, and D1. Lower values of D4, S2, and A2 were associated with increased risk of difficult laryngoscopic exposure, whereas higher D1 values contributed positively to risk. The direction of these effects was consistent with the multivariate logistic regression results, supporting the robustness of the model.

Previous studies on difficult laryngoscopic exposure in infants with PRS have primarily focused on two-dimensional airway measurements or isolated anatomical parameters. In contrast, the present study utilized quantitative 3D-CT assessment to integrate multiple structural dimensions, thereby providing a more comprehensive characterization of upper airway morphology. This multidimensional analysis further elucidated the synergistic contribution of tongue morphology, posterior pharyngeal wall approximation, and sagittal airway collapse to difficult laryngoscopic exposure. Mao et al. in a study of 69 PRS infants undergoing mandibular distraction osteogenesis (MDO), proposed a prediction rule based on the cross-sectional area at the epiglottic tip, reporting that values < 36.97 mm^2^ were indicative of difficult intubation (AUC = 0.81). Their findings emphasized that shortening of the tongue base–posterior pharyngeal wall distance and reduction in the epiglottic airway area were key anatomical risk factors (10). Similarly, Liu et al. (16) quantified pharyngeal cross-sectional areas in 96 PRS infants using an OpenCV-based approach and found a significant negative correlation between pharyngeal area and intubation difficulty (*r* = −0.54, *p* < 0.001). However, their analysis was limited to a single airway dimension and did not incorporate multivariable modeling (16). In addition, Lee et al. (3) reported that tongue length is directly associated with the severity of glossoptosis, identifying it as a critical quantitative marker of upper airway obstruction in PRS.

The final ExtraTrees model developed in this study requires only a small number of easily obtainable quantitative 3D-CT parameters to generate individualized predictions of difficult laryngoscopic exposure. Owing to its interpretability, reproducibility, and potential for visual output, the model offers a practical and objective tool for preoperative airway risk stratification in infants with PRS. With the continuous advancement of automated image segmentation and multimodal AI analytics, this model could be seamlessly integrated into radiology workstations or anesthesia information systems, may facilitate more objective and quantitative assessment of difficult airway risk. Such integration may provide anesthesiologists with more proactive and reliable decision support during high-risk procedures such as MDO.

Several limitations should be acknowledged. First, this was a single-center retrospective study; although the sample size exceeds prior studies in this field, multicenter prospective validation is needed to further establish generalizability. Second, CT-derived measurements may be influenced by patient positioning, respiratory motion, and image resolution. Standardized procedures were applied, but minor systematic errors cannot be excluded. Third, the model currently relies solely on structural 3D-CT metrics, without integration of automated AI segmentation, multimodal imaging, or clinical scores, which may further enhance predictive performance. Moreover, radiation exposure is an important consideration in infant populations. In this study, all CT scans were performed strictly based on clinical indications, and low-dose imaging protocols were applied in accordance with pediatric radiology standards to minimize radiation risk. The proposed model is designed to utilize routinely acquired clinical imaging data and does not require additional scans, thereby avoiding any incremental radiation burden. Future work may explore radiation-free alternatives, such as ultrasound or MRI, combined with AI-based approaches. Although standardized sedation was used to ensure image quality, it may induce mild pharyngeal muscle relaxation compared with the awake state, potentially affecting airway measurements. Future studies using dynamic imaging or different sedation protocols are warranted. In addition, this study included only infants with PRS undergoing mandibular distraction osteogenesis (MDO), representing more severe cases; therefore, the generalizability of the model to milder or non-surgical patients may be limited. The model is primarily intended for preoperative airway assessment in severe PRS. Future multi-center studies with broader cohorts are needed to validate its applicability. Therefore, this approach is most applicable in patients who have already undergone CT imaging as part of routine clinical evaluation, rather than as a universal screening tool.

## 5 Conclusion

In conclusion, this study systematically delineated the key anatomical determinants of difficult laryngoscopic exposure in infants with PRS through quantitative 3D-CT analysis and developed a prediction model. Among the evaluated algorithms, the ExtraTrees model demonstrated favorable performance in both the training cohort and a temporally independent validation cohort. These findings suggest that quantitative 3D-CT parameters may assist in preoperative risk assessment of difficult airway in infants with PRS. However, given the retrospective single-center design and limited sample size, further validation in larger, multicenter prospective studies is required before clinical implementation.

## Data Availability

The raw data supporting the conclusions of this article will be made available by the authors, without undue reservation.
